# Population Structure of Double-Stranded RNA Mycoviruses That Infect the Rice Blast Fungus *Magnaporthe oryzae* in Japan

**DOI:** 10.3389/fmicb.2020.593784

**Published:** 2020-10-28

**Authors:** Yuta Owashi, Mitsuhiro Aihara, Hiromitsu Moriyama, Tsutomu Arie, Tohru Teraoka, Ken Komatsu

**Affiliations:** ^1^ Laboratory of Plant Pathology, Graduate School of Agriculture, Tokyo University of Agriculture and Technology (TUAT), Fuchu, Japan; ^2^ Western Region Agricultural Research Center, National Agriculture and Food Research Organization, Fukuyama, Japan

**Keywords:** rice blast, mycovirus, *Pyricularia oryzae* (formerly *Magnaporthe oryzae*), *Magnaporthe oryzae virus 2*, *Magnaporthe oryzae* partitivirus 1, *Magnaporthe oryzae chrysovirus 1*, victorivirus, screening

## Abstract

Various viruses infect *Magnaporthe oryzae* (syn. *Pyricularia oryzae*), which is a well-studied fungus that causes rice blast disease. Most research has focused on the discovery of new viruses and the hypovirulence-associated traits conferred by them. Therefore, the diversity and prevalence of viruses in wild fungal populations have not been explored. We conducted a comprehensive screening of *M. oryzae* mycoviruses from various regions in Japan using double-stranded RNA (dsRNA) electrophoresis and RT-PCR assays. We detected three mycoviruses, Magnaporthe oryzae virus 2 (MoV2), Magnaporthe oryzae chrysovirus 1 (MoCV1), and Magnaporthe oryzae partitivirus 1 (MoPV1), among 127 of the 194 *M. oryzae* strains screened. The most prevalent virus was MoPV1 (58.8%), which often co-infected in a single fungal strain together with MoV2 or MoCV1. MoV2 and MoCV1 were found in 22.7 and 10.8% of strains, respectively, and they were usually distributed in different regions so that mixed-infection with these two mycoviruses was extremely rare. The predominance of MoPV1 in *M. oryzae* is supported by significant negative values from neutrality tests, which indicate that the population size of MoPV1 tends to increase. Population genetic analyses revealed high nucleotide diversity and the presence of phylogenetically diverse subpopulations among the MoV2 isolates. This was not the case for MoPV1. Furthermore, studies of a virus-cured *M. oryzae* strain revealed that MoV2 does not cause any abnormalities or symptoms in its host. However, a leaf sheath inoculation assay showed that its presence slightly increased the speed of mycelial growth, compared with virus-free mycelia. These results demonstrate that *M. oryzae* in Japan harbors diverse dsRNA mycovirus communities with wide variations in their population structures among different viruses.

## Introduction

Fungal viruses (mycoviruses) are widespread in a variety of fungal species. Since their first discovery in cultivated mushrooms (Hollings, 1962), many mycoviruses have been found in numerous fungal species. They are classified into three groups based on the compositions of their genomes: linear double-stranded RNA (dsRNA), linear positive- and negative-sense single-stranded RNA (ssRNA), and circular single-stranded DNA (ssDNA). Among these, mycoviruses with dsRNA genomes represent the majority, and they are classified into seven families: *Totiviridae* (a non-segmented genome of 4.6–7.0 kbp), *Amalgaviridae* (a non-segmented genome about 3.1–3.5 kbp), *Partitiviridae* (bisegmented genomes of 1.3–2.5 kbp), *Megabirnaviridae* (bisegmented genomes of 7.2–8.9 kbp), *Chrysoviridae* (3–7 segments of 2.4–3.6 kbp), *Quadriviridae* (four segments of 3.7–4.9 kbp), and *Reoviridae* (11–12 segments of 0.7–4.1 kbp; Ghabrial and Suzuki, 2009; Nibert et al., 2014; Ghabrial et al., 2015; Depierreux et al., 2016; Zhai et al., 2018).

One of the most widely used techniques for the detection of mycoviruses is extraction and gel electrophoresis of dsRNA elements (Morris and Dodds, 1979; Herrero et al., 2012; Okada et al., 2015). This technique is a relatively cheap and has an advantage of detecting most abundant mycoviruses including those having ssRNA or DNA genome, except for the viruses which may not accumulate abundant dsRNA as their replication intermediate during infection (Nerva et al., 2016). The numbers and sizes of dsRNA elements vary among dsRNA mycovirus species. Multiple dsRNA elements found in one fungal strain may be derived from multipartite viral genomes, mixed infections, or even from defective products of the viral RNA (Chiba et al., 2013b; Jiang et al., 2015). Mycoviruses generally have no extracellular phase; they are transmitted in a host-dependent, intracellular manner, such as through cell division, sporogenesis, or hyphal anastomosis (Ghabrial et al., 2015). Due to this exclusively intracellular lifestyle, it is likely that polymorphic dsRNA profiles and mycovirus phylogenic diversity are associated with specific geographical areas and host strains (Arakawa et al., 2002; Voth et al., 2006). However, this is not always the case (Schoebel et al., 2018; Wang et al., 2020).

Most mycoviruses infections cause no obvious symptoms in their fungal hosts. This could be explained by “the ancient infection hypothesis,” reflecting the long period of coevolution between fungal hosts and mycoviruses, and driving viruses into nonvirulence (Pearson et al., 2009). One good example is Rosellinia necatrix partitivirus 2, whose infection induces phenotypic alterations in an artificial host *Cryphonectria parasitica* but not in its natural host *R. necatrix* (Chiba et al., 2013b). In contrast, mycoviruses that attenuate virulence of their hosts are unusual. However, as exemplified by Cryphonectria hypovirus 1, viruses that induce so-called hypovirulence in their hosts can be used as biological control agents against plant pathogenic fungi (Milgroom and Cortesi, 2004; Rigling and Prospero, 2018). Other mycoviruses establish a relationship that is beneficial to the host fungus, even though they sometimes impair host fungal growth. For example, an *Alternaria alternata* strain with a high titer of Alternaria alternata chrysovirus 1 exhibits enhanced pathogenicity toward Japanese pear, and this is called hypervirulence (Okada et al., 2018). In many cases, it is difficult to comprehensively understand the complex interactions between mycoviruses and their hosts, because the effects of mycovirus infection might differ depending on environmental conditions and/or the plant cultivars that are infected (Aihara et al., 2018).

*Magnaporthe oryzae* (syn. *Pyricularia oryzae*) is an important fungus that is the causal agent of rice blast disease, and a number of mycoviruses have been reported to infect it. Among these are dsRNA viruses including Magnaporthe oryzae virus 1, 2, and 3 (MoV1, MoV2, and MoV3), which belong to the genus *Victorivirus* of the family *Totiviridae* (Yokoi et al., 2007; Maejima et al., 2008; Tang et al., 2015), Magnaporthe oryzae partitivirus 1 (MoPV1) in the family *Partitiviridae* (Du et al., 2016), and several isolates of Magnaporthe oryzae chrysovirus 1 (MoCV1-A, -B, -C, and -D) in the family *Chrysoviridae* (Urayama et al., 2010, 2012, 2014; Tang et al., 2015; Higashiura et al., 2019). In addition, some ssRNA viruses have been reported in this host: Magnaporthe oryzae virus A (MoVA) in the family *Tombusviridae* (Ai et al., 2016), Magnaporthe oryzae ourmia-like virus 1 and 4 and Pyricularia oryzae ourmia-like virus 1 to 3 (MOLV1, MOLV4, and PoOLV1–3), which are closely related to the ourmia-like viruses (Illana et al., 2017; Li et al., 2019; Ohkita et al., 2019), and Magnaporthe oryzae narnavirus 1 (MoNV1) in the family *Narnaviridae* (Lin et al., 2020). To date, these *M. oryzae* mycoviruses have been detected mainly in Asian countries, including China (MoV3, MoCV1-C, MoPV1, MoVA, MoLV4, and MoNV1), Japan (MoV1, MoV2, and MoCV1-D), and Vietnam (MoCV1-A and MoCV1-B). Among these mycoviruses, the MoCV1 isolates have been studied the most because they induce hypovirulence-associated traits in *M. oryzae*, including impaired mycelial growth and abnormalities in cell morphology (Urayama et al., 2010). MoLV4 was reported to be asymptomatic in its host (Li et al., 2019). However, most of these mycoviruses have not been studied to determine the symptoms they cause in their fungal host. Furthermore, their population structures in the natural environment have not been investigated.

In this study, we surveyed the mycovirus infections in Japanese *M. oryzae* strains, and analyzed their population structures. Screening of 194 *M. oryzae* strains in Japan for viruses by dsRNA electrophoresis and sequencing analyses revealed that the three mycoviruses MoV2 (a victorivirus), MoCV1 (a chrysovirus), and MoPV1 (a partitivirus) were present in the Japanese strains of *M. oryzae*, and their infection rates were quite variable depending on the regions, where the strains were found. Furthermore, this study examined whether MoV2 infection can affect the host phenotype. As a result, the elimination of a MoV2 isolate from its host strain revealed that MoV2 does not cause any abnormalities or symptoms in its host, but slightly promotes hyphal growth in infected plants.

## Materials and Methods

### Strains of *M. oryzae* and Culture Conditions

A total of 194 strains of *M. oryzae* were obtained from symptomatic rice leaves in various regions of Japan, including the Kyushu region (in southern Japan), the Hokuriku region (a coastal region in north-western Japan), and others. All strains were grown on potato dextrose agar (PDA) medium for 1 week, then mycelial plugs were incubated in YG broth [0.5% (w/v) yeast extract and 2% glucose] with shaking (65 r/min) for up to 2 weeks at 25°C. These mycelia were stored at −80°C until use.

### Purification and Detection of dsRNAs

Total nucleic acids were extracted, and dsRNA was subsequently purified using the micro-spin column method (Okada et al., 2015) with Cellulose D (Advantec, Japan). Fungal mycelium (approximately 0.1 g dry weight) was pulverized with 500 μl extraction buffer [2× STE (100 mM NaCl; 10 mM Tris-HCl, pH 8.0; 1 mM EDTA, pH 8.0) containing 0.1% (v/v) ß-mercaptoethanol and 1% (w/v) SDS], and then 500 μl phenol-chloroform-isoamyl alcohol (25:24:1) was added. The mixture was vortexed, centrifuged at 15,000 rpm for 5 min, and the supernatant was collected. The supernatant was mixed with ethanol (final concentration 16%) and loaded onto a spin column and centrifuged. After washing the column with 400 μl of wash buffer [1× STE containing 16% (v/v) ethanol; 15,000 rpm for 50 s, three times], the dsRNA solution was eluted with 350 μl of elution buffer (1× STE; 15,000 rpm for 1 min). The dsRNAs were concentrated by ethanol precipitation and dissolved in 20 μl nuclease-free water. The purified dsRNA solution (10 μl) was electrophoresed in a 1.0% (w/v) agarose gel (17 V, 18 h) and visualized by ethidium bromide staining to determine the sizes and numbers of dsRNA segments.

### Reverse Transcription (RT)-PCR and Direct Sequencing

One-step RT-PCR was carried out using purified dsRNA as the template. Virus-specific primer sets were designed based on the RNA-dependent RNA polymerase (RdRp) coding regions of MoCV1, MoPV1, MoV1, MoV2, and MoV3 (Supplementary Table S1). SuperScript™ III Reverse Transcriptase (Thermo Fisher Scientific, Witham, MA, United States) was used according to the manufacturer’s protocol, with a final reaction volume of 10 μl. Reactions were performed as follows: reverse transcription at 55°C for 20 min and 60°C for 10 min, followed by 94°C for 2 min, then 35 cycles of 94°C for 15 s, 55°C for 30 s, and 68°C for 45 s, then 68°C for 5 min. The amplified fragments were directly sequenced using the same primer sets in an ABI Prism 3130xl Genetic Analyzer (Thermo Fisher Scientific, Witham, MA, United States).

### Complete Genome Sequencing of the MoV2 Isolates

The dsRNAs extracted from *M. oryzae* strains from the Kyushu region, NHH12P-1, MZ4-12-2, and MZ13-12-1, were used as templates for cDNA synthesis using the PrimeScript™ first-strand cDNA Synthesis Kit (Takara Bio, Kusatsu, Japan) with MoV2-specific primers (Supplementary Table S1). Three overlapping PCR products were amplified using the KOD FX Neo polymerase (Toyobo, Osaka, Japan) with the primer sets Hind-T7-MoV2F/MoV2-2697R, MoV2-2657F/MoV2-detect R 4052–4073, and MoV2-detect F 3703–3725/Bam-MoV2R-re (Supplementary Table S1). The amplified PCR products were treated with 10× A-attachment mix (Toyobo, Osaka, Japan) and ligated into the pGEM T-Easy vector (Promega, Madison, United States), which was then used to transformation Hit-DH5α cells (RBC Bioscience, Taipei, Taiwan). Plasmid DNA was purified using the Wizard Plus SV Minipreps DNA Purification System (Promega, Madison, United States). Sequencing was conducted using both the universal primers (M13-M3 and M13-RV) and designed sequencing primers (Supplementary Table S1). The 5′- and 3′-terminal sequences of each dsRNA segment were determined using the SMARTer® RACE cDNA Amplification Kit (Clontech Laboratories, Inc., Mountain View, United States).

### Phylogenetic and Population Genetic Analyses

Total 35 isolates of MoV2 in Japan (29 isolates from the Kyushu region and six isolates from other regions) and 24 isolates of MoPV1 (23 isolates from the Kyushu region in Japan and an isolate from China) were used for phylogenetic and population genetic analyses. A partial RdRp-coding region of MoV2 (371 bp), which includes the conserved RdRp motifs characteristic of RdRps of the members of the family *Totiviridae* (Bruenn, 1993; Yokoi et al., 2007), as well as that of MoPV1 (466 bp), were analyzed. Total 32 isolates of MoV2 in Japan (29 isolates from the Kyushu region and 3 isolates from other regions) were also used for phylogenetic and population genetic analyses for 394 bp of partial coat protein (CP) coding domain. Nucleotide sequences were aligned using Clustal W (Thompson et al., 1994). Phylogenetic trees based on the nucleotide sequences were constructed by the Maximum Likelihood method using the MEGA 7.0 software (Kumar et al., 2016). Kimura’s two parameter model, assessed by the best-fit method, was applied for the calculation. Genetic parameters and neutrality tests were determined and carried out using DnaSP version 6.11 (Rozas et al., 2017). The rates of nonsynonymous (*d*_N_) and synonymous (*d*_S_) substitutions were estimated using the Pamilo-Bianchi-Li method, implemented in the MEGA 7.0 software.

### Fungal Growth and Inoculation Assays

Two *M. oryzae* strains: Ken 60-19, in which MoV2 was originally detected (Maejima et al., 2008), and the virus-cured strain Ken 60-19-VC, which was produced by single-spore isolation, were used for biological assays and pathogenicity tests. Morphologies and growth rates were assessed by culturing mycelial plugs (5 mm diameter) on PDA medium for 10 days at 25°C. The growth rate data were analyzed using a paired *t*-test with the software R version 3.4.1 (R Core Team, 2017). For microscopic observations of aerial mycelia, mycelial plugs were cultured in YG liquid medium at 25°C with shaking (65 r/min) for 1 week. The observations were performed using a light microscope with differential interference contrast optics (Olympus DP80).

Inoculation tests were conducted using seedlings of the rice variety Lijiangxintuanheigu (LTH). The seedlings were planted in plastic pots and were grown in a greenhouse. To induce conidiation, fungal strains were grown on solid oatmeal agar medium for 2 weeks at 25°C, and then aerial hyphae were removed and cultured under black light blue lamps for 3 days. The conidia were brushed off into distilled water.

For spray inoculations, conidia were suspended in distilled water containing 0.1% (v/v) Tween 20 to a final density of 1 × 10^5^ conidia/ml. The conidial suspension was sprayed on 3-week-old rice seedlings at a rate of 1 ml/individual. After 24 h of incubation in a humid box (24°C in the dark), the seedlings were placed in a growth chamber for 6 days at 25°C, and the disease was scored according to Hayashi et al. (2009).

For leaf sheath inoculations, 4-week-old leaf sheaths were cut into 4 cm segments, and the inner spaces were filled up with a 5 × 10^5^ conidia/ml suspension. After 48 h of incubation in a humid box (24°C in the dark), the sheath segments were trimmed to remove the sides and to expose the epidermal layer above the midvein. Lower midvein tissues were then sliced and observed using an Olympus CX41 microscope. Hyphal growth inside plants was scored with four grades as described by Namai et al. (1996): 1, no branched hyphae; 2, branched in a cell; 3, progression to other cells; and 4, filling more than two cells. The data for mycelial development were analyzed using the Wilcoxon rank-sum test with the R software.

## Results

### Detection of dsRNA Mycoviruses Infecting *M. oryzae* in Japan

**Figure 1:**
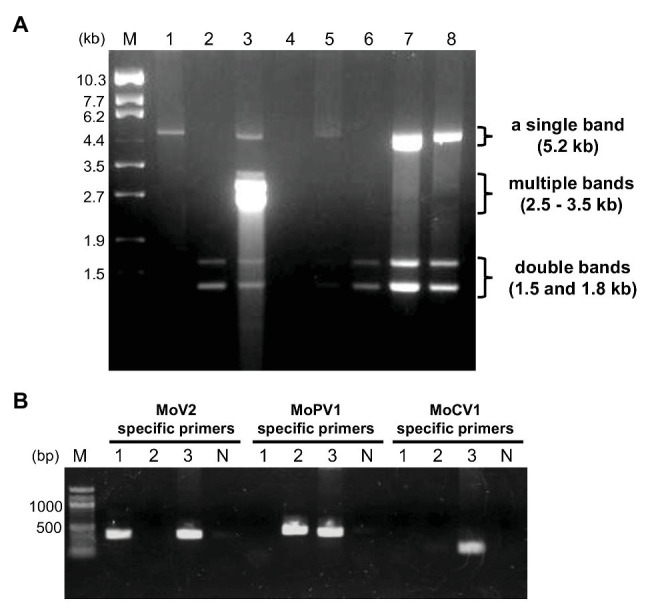
Detection of double-stranded RNA (dsRNA) mycoviruses infecting *Magnaporthe oryzae* by dsRNA electrophoresis and RT-PCR. **(A)** An example of an electrophoretic profile of dsRNAs from *M. oryzae* strains in Japan (lane 1, MZ19-12-2; lane 2, KMK12P-5; lane 3, APU10-199A; lane 4, MZ3-12-2; lane 5, MZ12-12-2; lane 6, MZ10-12-1; lane 7, NHH12P-1; and lane 8, KIO12P-11). Lane M, DNA size markers (λDNA digested with *Eco*T14 I). **(B)** Specific detection of three mycoviruses by RT-PCR. Lane numbers 1–3 correspond to the same *M. oryzae* strains as in **(A)**. Lane N, negative control with no DNA; lane M, 100 bp DNA ladder (New England BioLabs: N3231).

In order to identify dsRNA mycoviruses infecting Japanese *M. oryzae* strains, we purified dsRNA from fungal mycelia and performed electrophoresis. Among 194 strains, we found three types of dsRNA elements: a single band at about 5.2 kb, multiple bands ranging from 2.5 to 3.5 kb, and double bands at about 1.5 and 1.8 kb (Figure 1A). Based on the sizes and numbers of dsRNA elements in known *M. oryzae* mycoviruses, these three types of dsRNA elements represented the genome segments of MoV1/2/3, MoCV1, and MoPV1, respectively (Yokoi et al., 2007; Maejima et al., 2008; Urayama et al., 2010; Tang et al., 2015; Du et al., 2016).

We used virus-specific RT-PCR and partial sequencing to confirm the identities of the three dsRNA mycoviruses (Figure 1B). Among the *M. oryzae* strains tested (35 strains from Kyushu and other regions), all those carrying dsRNA elements of about 5.2 kb produced amplification products when tested with the MoV2-specific primers. No amplification products were obtained when the same set of strains were tested with MoV1- and MoV3-specific primers (data not shown). Direct sequencing of these amplified products showed that they exhibited over 91.9% nucleotide identity with the first reported MoV2 isolate (from strain Ken 60-19, Japan: GenBank Accession no. AB300379). They exhibited less than 54.3 and 64.4% identities, respectively, with the previously reported MoV1 (from strain TH 65-105, Japan: GenBank Accession no. AB176964) and MoV3 (from strain QSP5, China: GenBank Accession no. KP893140). Similarly, the strains with multiple bands ranging from 2.8 to 3.5 kb (two of the 35 tested strains) produced amplification products when tested with the MoCV1-specific primers, and those with double bands at about 1.5 and 1.8 kb (23 of the tested strains) produced amplification products when tested with the MoPV1-specific primers. Sequencing of these amplification products showed that they exhibited higher than 85.4 and 96.8% identities, respectively, with the previously obtained sequences of MoCV1 (from strain S-0412-II 1a, Vietnam: GenBank Accession no. AB560761) and MoPV1 (from strain NJ20, China: GenBank Accession no. KX119172). We concluded that *M. oryzae* strains collected in Japan could be infected with one or more of the three dsRNA mycoviruses MoV2, MoCV1, and MoPV1.

### Prevalence of dsRNA Mycoviruses in *M. oryzae* in Japan

**Figure 2:**
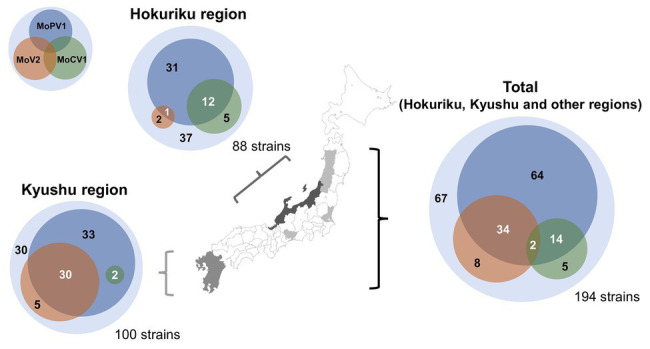
Frequencies of Magnaporthe oryzae virus 2 (MoV2), Magnaporthe oryzae partitivirus 1 (MoPV1), and Magnaporthe oryzae chrysovirus 1 (MoCV1) detected in *M. oryzae* from each area of Japan. Each inner circle indicates the number of *M. oryzae* strains infected with MoV2 (orange), MoPV1 (blue), and/or MoCV1 (green). Overlapping circles indicate multiple infections. The outer circles indicate the numbers of *M. oryzae* strains that had none of these mycoviruses.

We next performed a comprehensive investigation of the dsRNA mycoviruses in 194 Japanese *M. oryzae* strains. These included 100 strains from the Kyushu region (in southern Japan), 88 strains from the Hokuriku region (a coastal region in north-western Japan), and six strains from other regions. We found that that 127 of the 194 strains were infected with one or more of the three dsRNA mycoviruses (Figure 2 and Supplementary Table S2). All three mycoviruses were detected in strains from the Hokuriku region: 3/88 strains had MoV2, 17/88 strains had MoCV1, and 44/88 strains had MoPV1. Likewise, among strains from the Kyushu region, we detected MoV2 in 35/100 strains, MoCV1 in 2/100 strains, and MoPV1 in 65/100 strains. Among the six strains from other regions, all six had MoV2, two had MoCV1, and one had MoPV1. Some *M. oryzae* strains were co-infected with multiple mycoviruses. MoPV1 was often present with MoV2 and/or MoCV1; however, co-infections of MoV2 and MoCV1 were rare, and observed in only two cases (Figure 2).

### Phylogenetic Analysis of MoV2 and MoPV1

**Figure 3:**
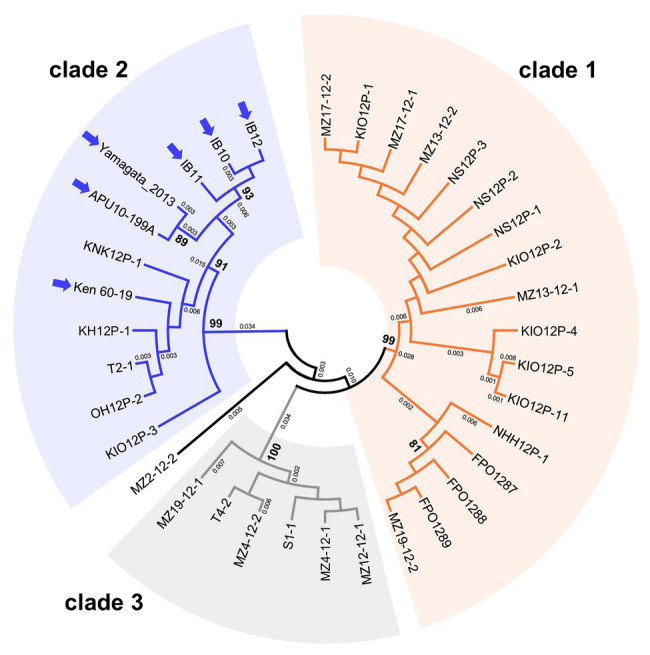
An unrooted phylogenetic tree, inferred by the Maximum Likelihood method based on the Kimura 2-parameter model (Kimura, 1980), was constructed using partial nucleotide sequences (371 bp) of the RNA-dependent RNA polymerase (RdRp) coding domain in 35 MoV2 isolates from Japan. Numbers at nodes represent bootstrap values calculated from 1,000 replicates, and smaller numbers indicate genetic distances. Bootstrap values less than 70% are not shown. Each MoV2 isolate is indicated by the name of its host *M. oryzae* strain. MoV2 isolates from other regions (see Supplementary Table S2) are indicated by arrows.

We performed phylogenetic analyses to examine the intraspecies population structures of MoV2 and MoPV1, which were the two major viruses found in our screening. For MoV2, the analysis was performed using partial nucleotide sequences (371 bp) from the RdRp coding domain. Among the 35 MoV2 isolates examined, there were three tentative clades with high bootstrap support (Figure 3). MoV2 isolates from the Kyusyu region were present in all three clades. On the other hand, all MoV2 isolates from other regions (shown by arrows in Figure 3) belonged to “clade 2,” along with some isolates from the Kyushu region. Almost the same result was shown in partial nucleotide sequences (394 bp) from the CP coding domain (Supplementary Figure S1). The phylogenetic tree revealed no clear clustering patterns based on geographic region or co-infection status within the Kyushu population (Supplementary Figure S2). However, some clustering based on geographical location was visible among the MoV2 isolates from other regions. The MoV2 isolates from two *M. oryzae* strains, APU10-199A, and Yamagata 2013 (both from northern Japan), and from three other *M. oryzae* strains, IB10, IB11, and IB12 (from the middle of the northeastern area of Japan), formed distinct subclades within ”clade 2” (Figure 3).

For the phylogenetic analysis of the MoPV1 isolates, we used 466 bp partial nucleotide sequences of the RdRp coding domain. Among the 24 isolates tested, there were no well-supported clusters (Supplementary Figure S3). The MoPV1 isolate (GenBank Accession no. KX119172) in the Chinese *M. oryzae* strain NJ20 was slightly separated from the Japanese isolates, although they all showed high sequence identities, with greater than 96.8% identity at the nucleotide level and greater than 97.4% identity at the amino acid level.

### Population Genetic Parameters and Neutrality Tests of MoV2 and MoPV1

The population genetic parameters for the two viruses based from partial sequence sets showed that the MoV2 isolates were more genetically variable than the MoPV1 isolates (Table 1). For example, the nucleotide diversity (π) and haplotype diversity (Hd) of RdRp coding domain among the MoV2 isolates (π = 0.04486, Hd = 0.928) were considerably higher than those of MoPV1 (π = 0.00397, Hd = 0.605). High nucleotide diversity and haplotype diversity were also shown in CP coding domain among the MoV2 isolates (π = 0.03837, Hd = 0.954). The *d*_N_/*d*_S_ ratios were less than 1 for both MoV2 and MoPV1 (Table 1), suggesting that both viruses underwent purifying selection. The similar nucleotide diversity and low *d*_N_/*d*_S_ ratios were calculated from full length of MoV2 sequences (Supplementary Table S3).

**Table 1:** Population genetic parameters for the partial RdRp and coat protein (CP) sequences of MoV2 and MoPV1.

Virus	Gene	*n*	Net sites	*S*	*h*	π	Hd	*d*_N_	*d*_S_	*d*_N_/*d*_S_
MoV2	RdRp	35	366	47	19	0.04486	0.928	0.0054	0.1703	0.0317
MoV2	CP	32	394	47	20	0.03837	0.954	0	0.1354	0
MoPV1	RdRp	24	466	16	7	0.00397	0.605	0.0009	0.0118	0.0763

n, number of isolates examined; Net sites, length of sequence (excluding sites with gaps and missing data); S, number of polymorphic sites; h, number of haplotypes; π, nucleotide diversity; Hd, haplotype diversity; *d*_N_, nonsynonymous rate; *d*_S_, synonymous rate.

Demographic forces on the MoV2 and MoPV1 populations were estimated using *D* statistics of Tajima (1989) and *D* and *F* statistics of Fu and Li (1993) (Table 2). These tests gave positive but not significant values for the MoV2 population and significantly negative values for the MoPV1 population. Positive values with these statistics indicate the occurrence of balancing selection, suggesting a decrease in population size, and negative values indicate a low frequency of polymorphisms and the occurrence of purifying selection, suggesting an increase in population size. The significantly negative values for the MoPV1 population suggest that their population size tends to increase.

**Table 2:** Neutrality test for partial RdRp and CP sequences of MoV2 and MoPV1.

Virus	Gene	*n* ^a^	Net sites^b^	Tajima’s *D*	Fu and Li’s *D*	Fu and Li’s *F*
MoV2	RdRp	35	366	1.58983 ns	0.43511 ns	0.97952 ns
MoV2	CP	32	394	1.08626 ns	−0.14516 ns	0.31312 ns
MoPV1	RdRp	24	466	−2.02511^*^	−2.94973^*^	−3.11718^*^

a Number of isolates.

b Length of sequence (excluding sites with gaps and missing data).

* *p* < 0.05.

### Sequence Properties of Three MoV2 Isolates

To examine whether the genome structures of the MoV2 isolates detected in this study are similar to those of the previously reported MoV2 isolates from *M. oryzae* strains Ken 60-19 (Maejima et al., 2008) and APU10-199A (Higashiura et al., 2019), we sequenced the entire MoV2 genomes from the *M. oryzae* strains NHH12P-1, MZ4-12-2, and MZ13-12-1, which all came from the Kyushu region. The complete sequences consisted of 5,194 or 5,197 nucleotides and contained two open reading frames (ORFs). BLAST searches^1^ revealed significant identities (>93.8%) between each of the three new MoV2 isolates and the isolates from Ken 60-19 and APU10-199A (Table 3). All the MoV2 isolates have the same lengths of 5′-untranslated region (275 nt) and 3′-untranslated region (63 nt). In all isolates, ORF1 has 2,367 nt, encoding a 788 aa coat protein. ORF2 encodes the RdRp; in the isolates from NHH12P-1 and MZ4-12-2, this gene consists of 2,493 nt encoding a protein of 830 aa, and in the isolate from MZ13-12-1, the gene has 2,496 nt, encoding a protein of 831 aa (Supplementary Table S4). In all three new isolates of MoV2, the termination codon of ORF1 overlaps the initiation codon of ORF2 in the tetranucleotide AUGA. This is also the case for the isolate from Ken 60-19, but in the isolate from APU10-199A, the ORFs overlap in an octonucleotide sequence, AUGAUUGA.

**Table 3:** Pairwise comparisons of complete nucleotide sequences between three tested MoV2 isolates and two reference isolates.

MoV2 tested isolate^a^	MoV2 reference isolates^a^	Identity (%)
NHH12P-1	Ken 60-19 APU10-199A	94.0 93.9
MZ4-12-2	Ken 60-19 APU10-199A	94.6 94.5
MZ13-12-1	Ken 60-19 APU10-199A	93.9 93.8

a The names are those of the host *M. oryzae* strains.

### A Comparison Between MoV2 Infected and Cured *M. oryzae* Strains

**Figure 4:**
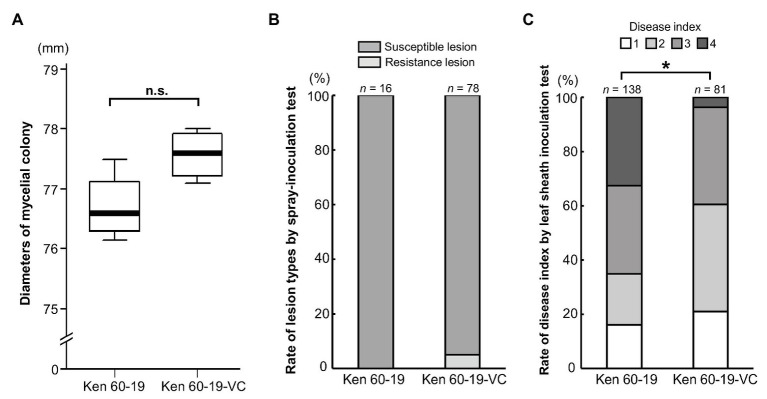
Comparison of the MoV2-infected *M. oryzae* strain Ken 60-19 and its corresponding virus-cured strain Ken 60-19-VC. **(A)** Diameters of mycelial colonies grown on potato dextrose agar (PDA) medium at 25°C for 10 days (*n* = 4). No significant differences were observed by the paired *t*-test (*p* = 0.0527). **(B)** Percentages of rice blast lesion types on the rice cultivar Lijiangxintuanheigu (LTH) after a spray-inoculation test, at 7 days post inoculation. **(C)** Percentage of each disease index score in LTH after a leaf sheath inoculation test, at 48 h post inoculation. Significant differences were found based on the Wilcoxon rank sum test (^*^ *p* = 0.0234).

Even though MoV2 is widely distributed in Japan, its effects on the host had not been examined. To investigate the effects of MoV2 infection on host virulence, we evaluated the biological characteristics of the *M. oryzae* strain Ken 60-19, which is solely infected with MoV2, and Ken 60-19-VC, which is an MoV2-cured variant of Ken 60-19 (Supplementary Figure S4). Mycelial growth rates on PDA medium were not significantly different between Ken 60-19 and Ken 60-19-VC (Figure 4A), and there were no obvious differences in pigmentation or morphology of the aerial mycelia (Supplementary Figures S5A,B). Similar lesions were observed after spray-inoculation tests, with 100% of the lesions in plants infected by Ken 60-19 being scored as susceptible, and 94.9% of those in plants infected by Ken 60-19-VC being scored as susceptible (Figure 4B). However, the results of the leaf sheath inoculation tests demonstrated that the disease index scores were significantly different between plants infected with Ken 60-19 and those infected with Ken 60-19-VC (Figure 4C). In the Ken 60-19-infected plants, mycelial development inside the plant cells tended to be faster than in the plants infected with Ken 60-19-VC.

## Discussion

In previous studies, three dsRNA viruses, MoV1, MoV2, and MoCV1, were found in *M. oryzae* strains from Japan (Yokoi et al., 2007; Maejima et al., 2008; Higashiura et al., 2019). In this study, we performed a comprehensive screening of dsRNA mycoviruses from *M. oryzae* for the first time, and detected MoV2 and MoCV1, but not MoV1, in *M. oryzae* in Japan. MoV1 and MoV2 were among the earliest mycoviruses to be identified in Japan, and were first reported almost half a century ago (Yamashita et al., 1971). It is possible that the MoV1 was not detected in this study because of its low prevalence and/or its localized distribution. The rates of infection with mycoviruses vary widely among species and regions, ranging from a few percent to over 90%. For example, Botrytis cinerea mymonavirus 1 (BcMyV1) was detected in only 0.8% of the tested *B. cinerea* strains in China (Hao et al., 2018). Schoebel et al. (2017) also reported a low prevalence (1.1%) of Hymenoscyphus fraxineus mitovirus 1 (HfMV1) infection in *H. fraxineus* in Japan, whereas its prevalence was much higher in Europe (78.7%), especially in Lithuania (92.9%). We mainly screened *M. oryzae* strains from the Kyushu and Hokuriku regions in Japan, and more extensive screening may result in the re-detection of MoV1. It is also possible that the MoV1 population has diminished over the past half-century. There have been few studies of the long-term population dynamics of mycoviruses; however, some studies have suggested that their populations do change over time (Vainio et al., 2013; Bryner et al., 2014). Thus, the mycovirus population sizes in Japanese *M. oryzae* may have changed since MoV1 was first detected.

In addition to MoV2 and MoCV1, which have been reported from Japan previously, we also detected MoPV1 for the first time in Japan. The partial RdRp sequences showed over 96.8% identity with the reference sequence of a Chinese isolate (Du et al., 2016). Since the MoCV1 isolates has been found worldwide, including in Vietnam (MoCV1-A, -B; Urayama et al., 2010, 2014), China (MoCV1-C; Tang et al., 2015) and Japan (MoCV1-D; Higashiura et al., 2019), further investigations might lead to the detection of MoPV1 in other countries. In our electrophoresis analysis, we also found unclassified dsRNAs patterns other than those of MoV2, MoCV1, and MoPV1 (unpublished data). It is thus possible that we might discover new dsRNA mycoviruses from *M. oryzae* in Japan, by conducting next-generation sequencing analysis (e.g., Komatsu et al., 2016).

Among the 194 strains of *M. oryzae* tested in the present study, 127 strains (65.5%) were infected with MoV2, MoCV1, and/or MoPV1. The most prevalent of these three mycoviruses was MoPV1 (58.8%), followed by MoV2 (22.7%), and the least prevalent was MoCV1 (10.8%). These differences may be mainly explained by the different rates of vertical transmission with each mycovirus, because they have no extracellular phase and are transmitted only *via* host-dependent intracellular mechanisms (Ghabrial et al., 2015). A recent study revealed that the transmission rate of MoCV1-A *via* secondary conidia, whose production is induced by black light blue lamps after harvesting primary conidia, in the host strain S-0412-II 1a is about 80% (Aihara et al., 2018). Similar, less efficient transmission through conidia was reported for MoCV1-D in the *M. oryzae* strain APU10-199A (Higashiura et al., 2019). In contrast to MoCV1, MoV2 and MoPV1 are generally stably maintained in conidia, as revealed by single-spore isolation of the strain APU10-199A, which was co-infected with MoCV1, MoV2, and MoPV1 (Higashiura et al., 2019). MoCV1 may have reduced opportunities for vertical transmission, because infection with MoCV1 can severely decrease the production of conidia by the host fungus. In fact, a MoCV1-B-infected *M. oryzae* strain is unable to form conidia (Urayama et al., 2014). However, we cannot exclude the possibility that factors other than vertical transmission rates influence the prevalence of each mycovirus species. Indeed, although MoCV1 was the least prevalent among the three mycoviruses we examined, it was detected more frequently than MoV2 in the Hokuriku region (Figure 2). Aihara et al. (2018) suggested that MoCV1 could increase the fitness of its host *M. oryzae*, especially in specific rice lines containing a particular *R* gene, based on their finding that MoCV1 converted the host fungus from avirulent to virulent in the specific rice line. We could not carry out pathogenicity tests of the fungal strains screened in this study with different rice varieties. However, the improved fitness of the *M. oryzae* strain Ken 60-19 when infected with MoV2 suggests that this effect of MoV2 could contribute to its differing spatial distribution between the Kyushu and the Hokuriku regions.

We observed relatively high nucleotide and haplotype diversities in MoV2 compared with MoPV1 (Table 1). Based on the analysis of nonsynonymous and synonymous substitution rates (Table 1 and Supplementary Table S3), this high genetic diversity appeared to be mainly shaped by purifying selection, which has often been detected in genes of other mycoviruses (e.g., Botella et al., 2012; Schoebel et al., 2017). Moreover, although the MoV2 isolates from the Kyusyu region can be separated into three clades in the phylogenetic tree (Figure 3), we did not find any correlations related to the spatial distribution of MoV2 within the Kyushu region because the distribution areas of each clade were overlapped, and different clades of MoV2 were sometimes detected from same location (Supplementary Figure S2). Higashiura et al. (2019) reported that MoV2 is rarely transmitted horizontally *via* hyphal fusion, in contrast to their extremely high vertical transmission rates through conidia. These results suggested that the MoV2 isolates in each clade evolved independently of those in the other clades and were distributed throughout the Kyushu region as their host *M. oryzae* has spread (Voth et al., 2006; Bryner et al., 2012). Therefore, the distribution of MoV2 is likely driven by the virulence-dependent distribution of its host, which changes depending on which rice cultivars, possessing different resistance genes, are grown. Considering that the combination of *M. oryzae* races and rice cultivars determines the distribution of MoV2, its patterns of diversity could be formed independently of geographical barriers. This hypothesis can also explain the difference in prevalence of MoV2 between the Kyushu and Hokuriku regions, because the composition of *M. oryzae* races differs between these regions (Kawasaki-Tanaka et al., 2016). On the other hand, we found that the phylogenetic relationships among some MoV2 isolates were closely correlated with their spatial distributions. Therefore, we cannot rule out the possibility that neutral mutations have been fixed randomly in some MoV2 populations. For example, the MoV2 isolates from the APU10-199A and Yamagata 2013 strains, both from the northern part of Japan, and those from IB10, IB11, and IB12 from the middle northeastern part of Japan formed two distinct subclades within clade 2. Moreover, all the MoV2 isolates other than those from the Kyushu region belong to the same clade. This might indicate some geographical clustering of the MoV2 populations on a large scale. However, our sample sizes other than from the Kyushu region were not large enough to confirm this assumption. Further experiments with more extensive screening combined with genetic analyses of both MoV2 and its host would shed further light on the population structure and evolutionary history of MoV2 in Japan.

The extremely low genetic diversity among the MoPV1 isolates implies that their population emerged recently, or that they have been subjected to strong bottlenecks. Furthermore, the detection of MoPV1 in two countries (China and Japan), its high prevalence in Japan, and its frequent co-infection with other mycoviruses (Figure 2) suggest that its population size is increasing. This notion is supported by the significant negative values in the neutrality test (Table 2).

We determined the complete nucleotide sequences of three MoV2 isolates from the Kyushu region and found that they all had two ORFs overlapping within the tetranucleotide AUGA (Supplementary Table S4). This pattern is conserved in many other victoriviruses (Ghabrial and Nibert, 2009; Li et al., 2011), and is present in the MoV2 isolate from Ken 60-19 (Maejima et al., 2008) but not in the isolate from APU10-199A (Higashiura et al., 2019). In the isolate from APU10-199A, the two ORFs overlap within the octonucleotide AUGAUUGA. Although the overlapping of two ORFs in an octonucleotide sequence is observed in some other victoriviruses (e.g., Park et al., 2005), to our knowledge, this is the first example of two overlapping patterns in one species in the genus *Victorivirus*. Despite the difference in overlapping lengths, the complete nucleotide sequences of the three MoV2 isolates determined in this study were all highly similar (ranging in identity from 93.8 to 94.6%) to those of the MoV2 isolates from both Ken 60-19 and APU10-199A (Table 3). All five isolates use the opal codon (UGA) as the stop codon in their overlapped sequences. Future research will address the relationships between the types of overlapping sequences and the phylogeny within each mycovirus species.

Almost all victoriviruses infect their natural hosts without any apparent symptoms; examples include Rosellinia necatrix victorivirus 1 and Alternaria alternata victorivirus 1 (Chiba et al., 2013a; Jamal et al., 2019). Exceptions include Helminthosporium victoriae virus 190S, which induces disease symptoms in its natural host *H. victoriae* (Xie et al., 2016). As with most other victoriviruses studied thus far, MoV2 infection did not induce any obvious symptoms in its *M. oryzae* host, nor did it significantly change the fungal pathogenicity in rice plants (Figures 4A,B and Supplementary Figures S5A,B). Interestingly, however, our leaf sheath inoculation test showed that the MoV2-infected *M. oryzae* tended to show increased mycelial growth within rice cells, as compared with the MoV2-cured fungus (Figure 4C). The *M. oryzae* strains TH 65-105 and Ken 60-19, in which MoV1 and MoV2 were originally detected, produced larger amounts of the phytotoxic secondary metabolite pyriculol than other tested strains (Sato, 1978). Moreover, high concentrations of pyriculol have been shown to cause increased mycelial growth of *M. oryzae* (Namai et al., 1996). Although the causal relationship between MoV2 infection and increased pyriculol production is unknown, it is possible that the larger amount of pyriculol due to MoV2 infection in Ken 60-19 lead to its faster mycelial growth compared with its MoV2-cured variant.

## Data Availability Statement

The datasets presented in this study can be found in online repositories. The names of the repository/repositories and accession number(s) can be found at https://www.ddbj.nig.ac.jp/, LC573905-LC573960 and LC586101-LC586127.

## Author Contributions

TT collected the materials and designed the study. YO and MA performed the experiments with academic and technical assistance from TA. HM contributed to the DNA extractions and the virus-cured assays. YO and KK analyzed the data and wrote the first draft of the manuscript. All authors critically reviewed the manuscript and approved the final submission.

### Conflict of Interest

The authors declare that the research was conducted in the absence of any commercial or financial relationships that could be construed as a potential conflict of interest.

## Footnotes

^1^https://blast.ncbi.nlm.nih.gov/Blast.cgi
