# Supplementary material for: Population Structure of Double-Stranded RNA Mycoviruses That Infect the Rice Blast Fungus Magnaporthe oryzae in Japan
Source: Front Microbiol. 2020 Oct 28;11:593784. doi: 10.3389/fmicb.2020.593784 (PMC7664462; doi:10.3389/fmicb.2020.593784)
Supplement: Supplementary file 1 [file Data_Sheet_1.docx]

Supplementary Material


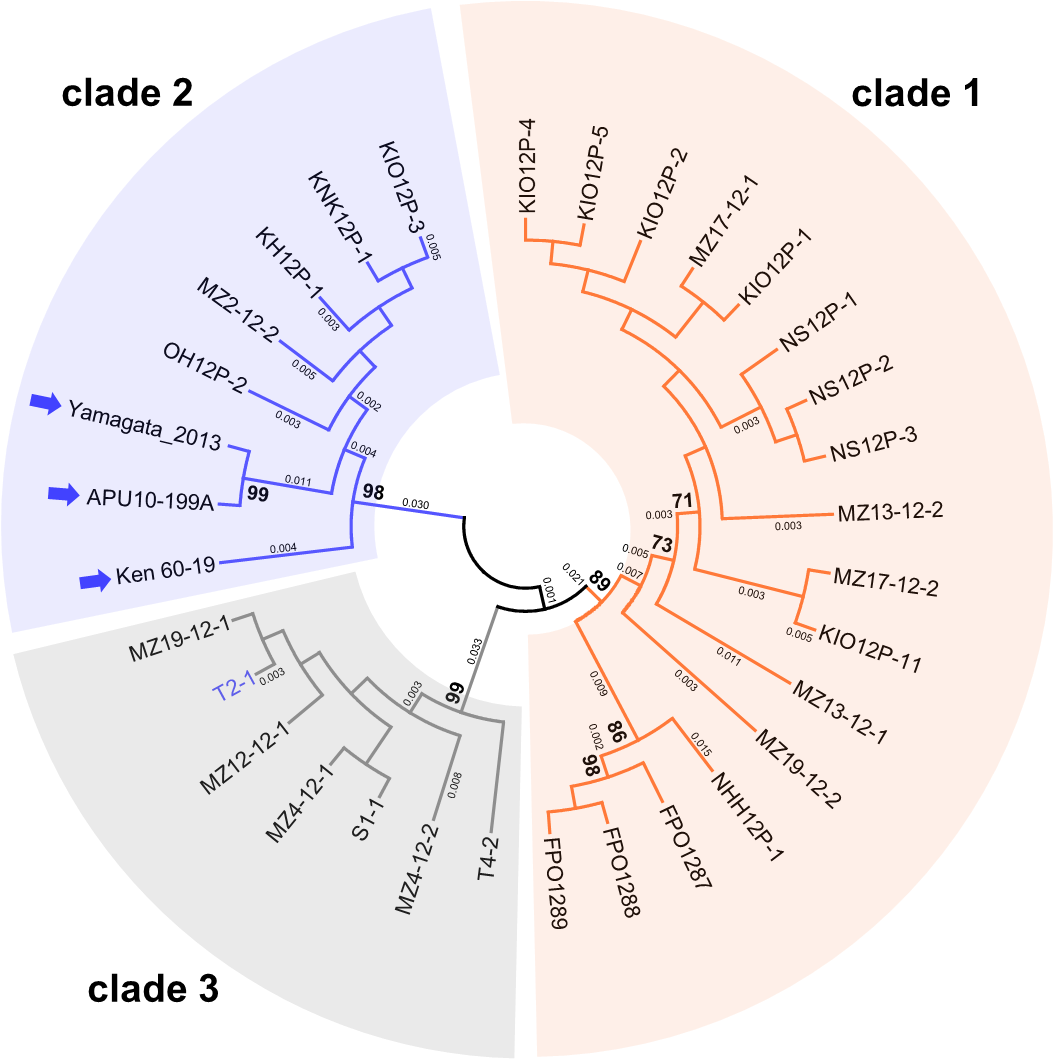


**Supplementary Figure S1.** An unrooted phylogenetic tree, inferred by the Maximum Likelihood method based on the Kimura 2-parameter model (Kimura, 1980), was constructed using partial nucleotide sequences (394 bp) of the CP coding domain in 32 MoV2 isolates from Japan. Numbers at nodes represent bootstrap values calculated from 1000 replicates, and smaller numbers indicate genetic distances. Bootstrap values less than 70% are not shown. Each MoV2 isolate is indicated by the name of its host *M. oryzae* strain. MoV2 isolates from outside the Kyusyu region are indicated by arrows. The blue text indicates the MoV2 isolate that belong to different clade from the phylogenetic tree of the RdRp coding domain in **Figure 3**.


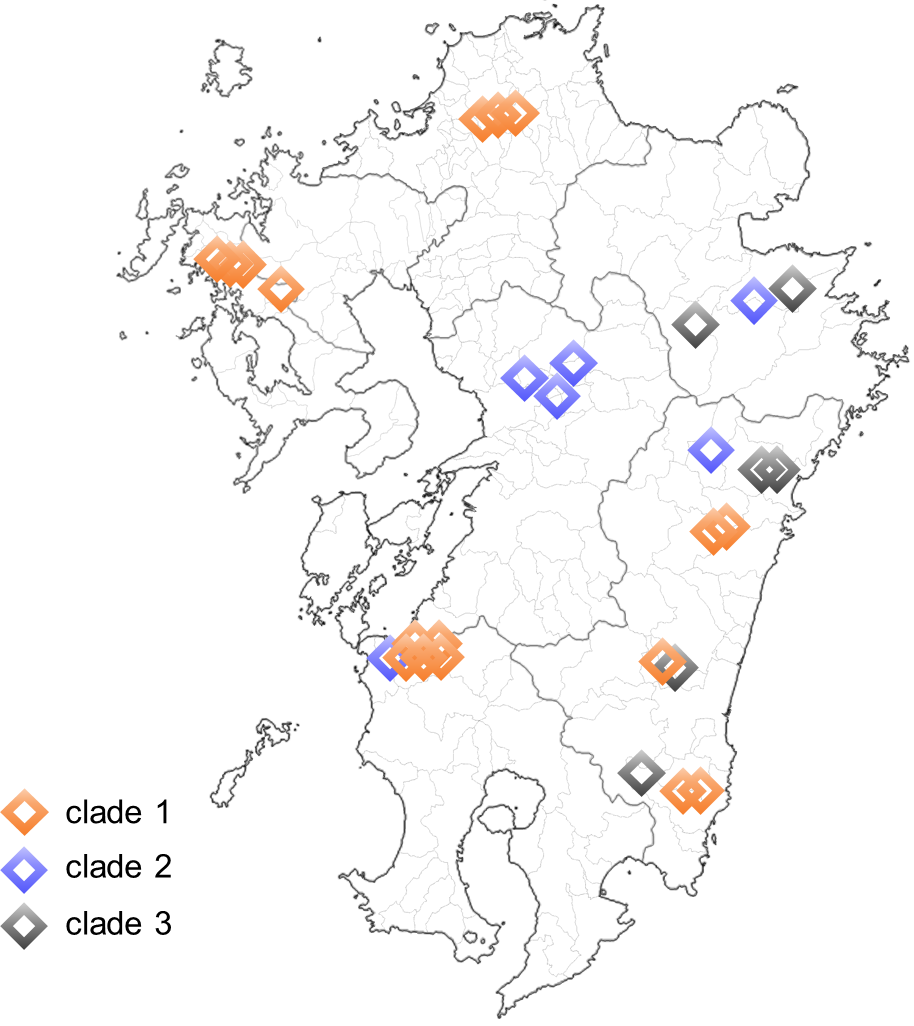


**Supplementary Figure S2.** Distribution of MoV2 in the Kyushu region. Each rhomb indicates location of MoV2 detected and each color indicates the clade of MoV2 based on phylogenetic tree of partial RdRp coding domain in **Figure 3**. The overlap of symbols indicates MoV2 from same location.


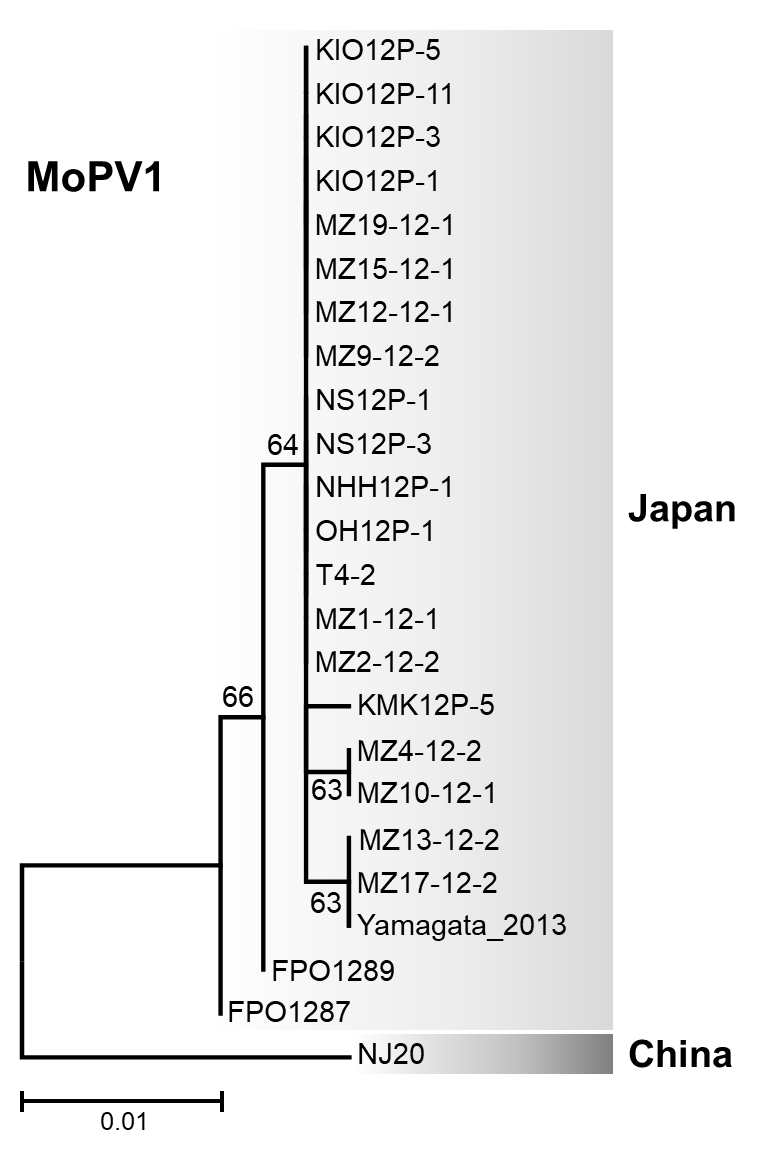


**Supplementary Figure S3.** An unrooted phylogenetic tree, inferred by the Maximum Likelihood method based on the Kimura 2-parameter model (Kimura, 1980), was constructed using partial nucleotide sequences (466 bp) of the RdRp coding domain in 23 MoPV1 isolates from Japan, and one sequence from China isolate. Numbers at nodes represent bootstrap values calculated from 1,000 replicates. Each MoPV1 isolate is indicated by the name of its host *M. oryzae* strain.


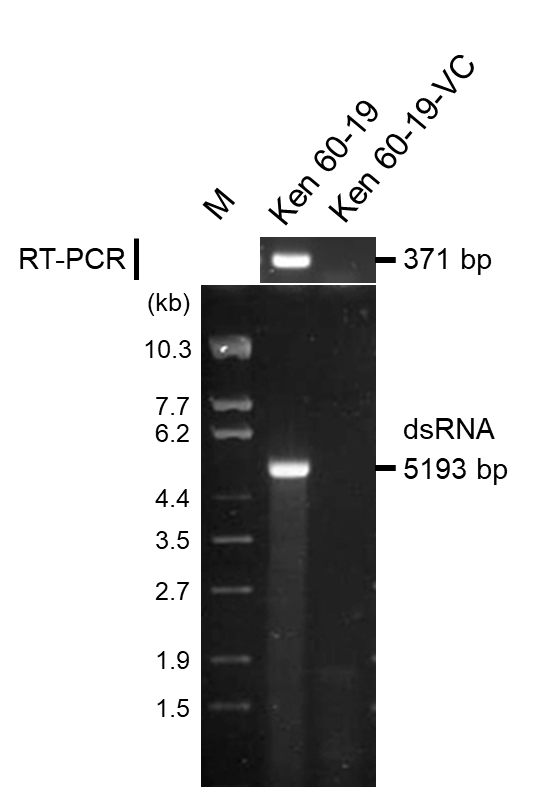


**Supplementary Figure S4.** Detections of MoV2 in *M. oryzae* strains Ken 60-19 and Ken 60-19-VC. Upper panel shows RT-PCR amplification using MoV2-specific primers. Lower panel shows pattern of agarose gel electrophoresis of dsRNA purified from each strain. Lane M, DNA size markers (λDNA digested with *Eco*T14 I).


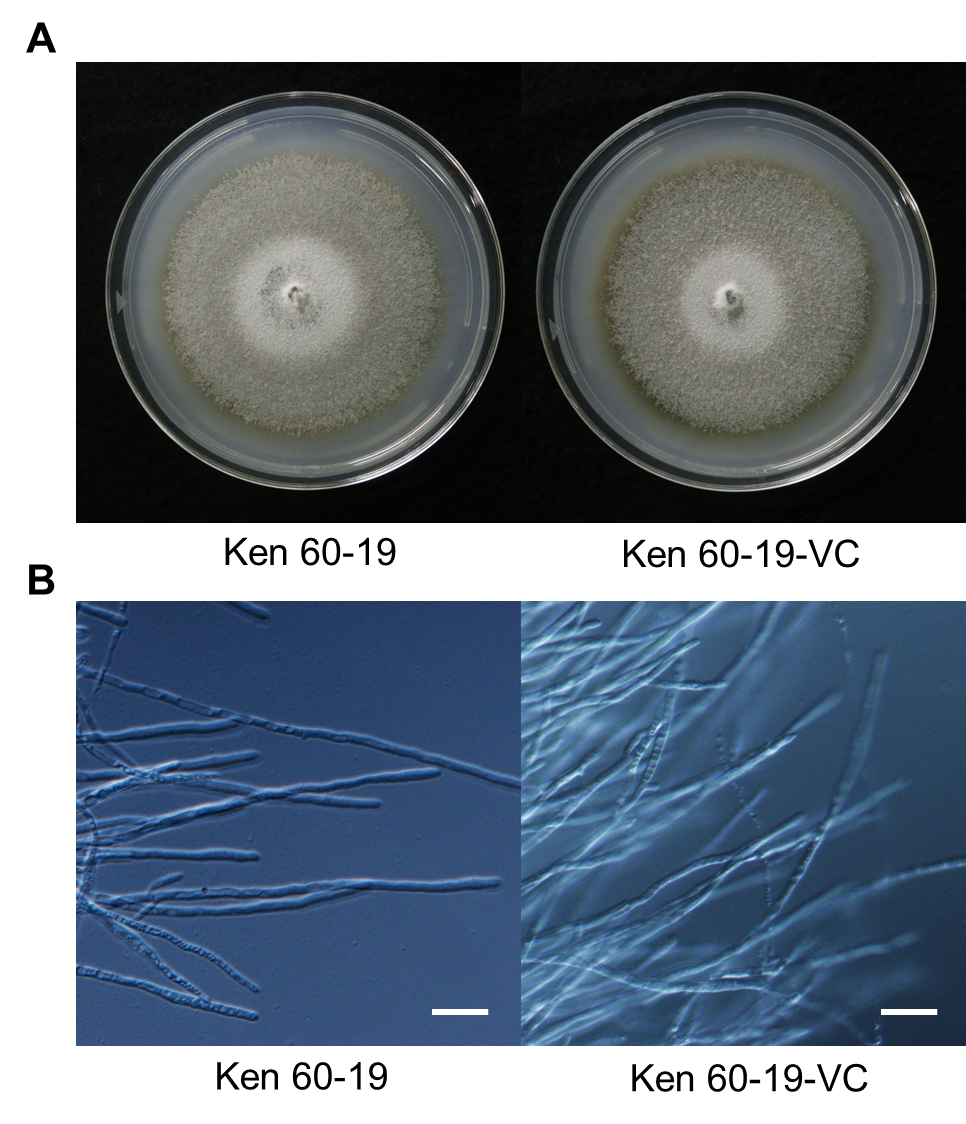


**Supplementary Figure S5.** Morphological characteristics of *M. oryzae* strains Ken 60-19 and Ken 60-19-VC. **(A)** Colony on PDA culture at 25°C for 10 days. **(B)** Aerial mycelia in YG liquid culture at 25°C for 1 week. Bars show 20 μm.

**Supplementary Table S1.** Oligonucleotide primers used in this study.

| Primer name | Oligonucleotide sequence (5'-3') | Template (target position)^a^ | Purpose^b^ |
| --- | --- | --- | --- |
| MoV1-detect F | AAGACGCGTGCGATCTTTGCTT | MoV1 (3877**–**3899) | Det. |
| MoV1-detect R | GGTGCCCGGACATGAGCGTAC | MoV1 (4226**–**4246) | Det. |
| MoV2-detect F 3703–3725 | AAGACCAGAGCAATCTTTGCCT | MoV2 (3703**–**3725) | Det., Seq., PCR |
| MoV2-detect R 4052–4073 | CGGTGGCCACTCATCAGAGTAC | MoV2 (4052**–**4073) | Det., Seq., PCR, cDNA synthesis |
| MoV2-CP_886F | TACCAACCAGCCAATCATCC | MoV2 (886–905) | Seq. |
| MoV2-CP_1279R | GACTGTCGGGAACAGACCAC | MoV2 (1260–1279) | Seq. |
| MoV3-3709F | CACTCAGTTACCTAGCTTTCG | MoV3 (3709**–**3729) | Det. |
| MoV3-4049R | ATGGCCGCTCATTAAGGTCC | MoV3 (4030**–**4049) | Det. |
| MoCV1C-ID9-F3 | CGGTGTGATGCTGGACTAC | MoCV1-D segment 1 (2080**–**2098) | Det. |
| MoCV1C-ID9-B3 | GCAACTCACCGTCGTACTC | MoCV1-D segment 1 (2246**–**2264) | Det. |
| MoPV-RdRp-F | ATCTTACCACCGTGCATTGC | MoPV1 segment1 (425**–**444) | Det., Seq. |
| MoPV-RdRp-R | ACTCCAGTCCATGGTTAGCTC | MoPV1 segment1 (870**–**890) | Det., Seq. |
| Hind-T7-MoV2F | TAATACGACTCACTATAG GATAGAAAACGAAGAATCGAAAGTTTCCC | MoV2 (1**–**29) | cDNA synthesis, PCR |
| MoV2-2697R | CAGGTATTGGCCCAAGGAG | MoV2 (2679**–**2697) | PCR |
| MoV2-2657F | CCACAAGAGCTAAAGCAAC | MoV2 (2657**–**2675) | PCR |
| Bam-MoV2R-re | GTAAAACGACGGCCAGTGAATTCGGATCCGCATTATGGGCCCCGTG | MoV2 (5177**–**5193) | cDNA synthesis, PCR |
| MoV2-1887F | TCTCGTCGTATCTGTGGCG | MoV2 (1887**–**1905) | Seq. |
| MoV2-1140R | AAGGCACGGAGGGATTGGTT | MoV2 (1121**–**1140) | Seq. |
| MoV2-2008R | CGCGTTGAAATCGTTATCCCA | MoV2 (1988**–**2008) | Seq. |
| MoV2-3155F | TCTACGGCTACGGATTGAGT | MoV2 (3155**–**3174) | Seq. |
| MoV2-3358R | CCTCCTCGCGCAAGTCGATT | MoV2 (3339**–**3358) | Seq. |
| MoV2-4076F | GCACAACCTACATCAACAGC | MoV2 (4076**–**4095) | Seq. |
| MoV2-229R | CGTTGGTATCAACGGAACCCTCCTCC | MoV2 (204**–**229) | RACE |
| MoV2-4939F | TGACAGGATCGCAGCTACGCCGC | MoV2 (4939**–**4961) | RACE |

^a^The reference sequences registered in GenBank (MoV1, AB176964; MoV2, AB300379; MoV3, KP893140; MoCV1-D, LC432338; MoPV1, KX119172) were used, respectively.

^b^Det., mycovirus detection; Seq., sequencing.

**Supplementary Table S2.** Mycoviruses screening from *M. oryzae* strains in Japan by dsRNA electrophoresis assay.

|  | **Strain**^a^ | **Viruses**^b^ | | | **Year**^c^ | **Location, prefecture**^d^ | **Accession no.**^e^ | |
| --- | --- | --- | --- | --- | --- | --- | --- | --- |
|  |  | **MoV2** | **MoCV1** | **MoPV1** |  |  | **MoV2** | **MoPV1** |
| **Kyushu region** | | | | | | | | |
| 1 | FPO1229 | − | − | − | 2012 | Onga, Onga, Fukuoka | - | - |
| 2 | FPO1233 | − | − | + | 2012 | Nakama, Fukuoka | - | - |
| 3 | FPO1237 | − | − | + | 2012 | Yamakawa, Miyama, Fukuoka | - | - |
| 4 | FPO1248 | − | − | − | 2012 | Kama, Fukuoka | - | - |
| 5 | FPO1249 | − | − | + | 2012 | Kama, Fukuoka | - | - |
| 6 | FPO1250 | − | − | − | 2012 | Kama, Fukuoka | - | - |
| 7 | FPO1273 | − | − | − | 2012 | Uchikoshi, Itoda, Tagawa, Fukuoka | - | - |
| 8 | FPO1274 | − | − | − | 2012 | Uchikoshi, Itoda, Tagawa, Fukuoka | - | - |
| 9 | FPO1287 | + | − | + | 2012 | Iizuka, Fukuoka | LC573908, LC586101 | LC573938 |
| 10 | FPO1288 | + | − | + | 2012 | Iizuka, Fukuoka | LC573909, LC586102 | - |
| 11 | FPO1289 | + | − | + | 2012 | Iizuka, Fukuoka | LC573910, LC586103 | LC573939 |
| 12 | FPO1290 | − | − | − | 2012 | Nomachi, Chikuzen, Asakura, Fukuoka | - | - |
| 13 | FPO1291 | − | − | − | 2012 | Nomachi, Chikuzen, Asakura, Fukuoka | - | - |
| 14 | OS98L-1 | − | − | + | 1998 | Sari, Ouchi, Karatsu, Saga | - | - |
| 15 | OS98L-2 | − | − | + | 1998 | Sari, Ouchi, Karatsu, Saga | - | - |
| 16 | OS98L-3 | − | − | + | 1998 | Sari, Ouchi, Karatsu, Saga | - | - |
| 17 | YM98L-1 | − | − | + | 1998 | Yamato, Saga, Saga | - | - |
| 18 | YM98L-2 | − | − | + | 1998 | Yamato, Saga, Saga | - | - |
| 19 | YM98L-3 | − | − | + | 1998 | Yamato, Saga, Saga | - | - |
| 20 | FSK98L-1 | − | − | − | 1998 | Fuji, Saga, Saga | - | - |
| 21 | FSK98L-2 | − | − | − | 1998 | Fuji, Saga, Saga | - | - |
| 22 | FSK98L-3 | − | − | − | 1998 | Fuji, Saga, Saga | - | - |
| 23 | MK98L-1 | − | − | − | 1998 | Mitsuse, Saga, Saga | - | - |
| 24 | MK98L-2 | − | − | + | 1998 | Mitsuse, Saga, Saga | - | - |
| 25 | MK98L-3 | − | − | + | 1998 | Mitsuse, Saga, Saga | - | - |
| 26 | NS12P-1 | + | − | + | 2012 | Sasebo, Nagasaki | LC573911, LC586104 | LC573940 |
| 27 | NS12P-2 | + | − | + | 2012 | Sasebo, Nagasaki | LC573912, LC586105 | - |
| 28 | NS12P-3 | + | − | + | 2012 | Sasebo, Nagasaki | LC573913, LC586106 | LC573941 |
| 29 | NHH12P-1 | + | − | + | 2012 | Hiekoba, Hasami, Higashisonogi, Nagasaki | LC573905 | LC573942 |
| 30 | NHH12P-2 | + | − | + | 2012 | Hiekoba, Hasami, Higashisonogi, Nagasaki | - | - |
| 31 | NHH12P-3 | − | − | + | 2012 | Hiekoba, Hasami, Higashisonogi, Nagasaki | - | - |
| 32 | NHT12P-1 | − | − | + | 2012 | Takebeta, Hasami, Higashisonogi, Nagasaki | - | - |
| 33 | NHT12P-2 | − | + | + | 2012 | Takebeta, Hasami, Higashisonogi, Nagasaki | - | - |
| 34 | NHT12P-3 | − | + | + | 2012 | Takebeta, Hasami, Higashisonogi, Nagasaki | - | - |
| 35 | NUA12P-1 | − | − | + | 2012 | Unzen, Azuma, Nagasaki | - | - |
| 36 | NUA12P-2 | − | − | + | 2012 | Unzen, Azuma, Nagasaki | - | - |
| 37 | NUA12P-3 | − | − | + | 2012 | Unzen, Azuma, Nagasaki | - | - |
| 38 | KSH12P-1 | − | − | − | 2012 | Hayashibaru, Shichijo, Kikuchi, Kumamoto | - | - |
| 39 | KSH12P-2 | − | − | − | 2012 | Hayashibaru, Shichijo, Kikuchi, Kumamoto | - | - |
| 40 | YKO12P-1 | − | − | + | 2012 | Oda, Kikuka, Yamaga, Kumamoto | - | - |
| 41 | YKO12P-2 | − | − | + | 2012 | Oda, Kikuka, Yamaga, Kumamoto | - | - |
| 42 | YKS12P-1 | − | − | − | 2012 | Shiimochi, Kahoku, Yamaga, Kumamoto | - | - |

|  | **Strain** | **Viruses** | | | **Year** | **Location, prefecture** | **Accession no.** | | |
| --- | --- | --- | --- | --- | --- | --- | --- | --- | --- |
|  |  | **MoV2** | **MoCV1** | **MoPV1** |  |  | **MoV2** | **MoPV1** | |
| 43 | YKS12P-2 | − | − | + | 2012 | Shiimochi, Kahoku, Yamaga, Kumamoto | - | - |  |
| 44 | YKY12P-1 | − | − | + | 2012 | Shicho, Kahoku, Yamaga, Kumamoto | - | - |  |
| 45 | YKY12P-2 | − | − | + | 2012 | Shicho, Kahoku, Yamaga, Kumamoto | - | - |  |
| 46 | KNK12P-1 | + | − | + | 2012 | Wabu, Koshi, Kumamoto | LC573914, LC586107 | - |  |
| 47 | KNK12P-2 | − | − | − | 2012 | Wabu, Koshi, Kumamoto | - | - |  |
| 48 | OH12P-1 | − | − | + | 2012 | Haizuka, Ozu, Kikuchi, Kumamoto | - | LC573943 |  |
| 49 | OH12P-2 | + | − | + | 2012 | Haizuka, Ozu, Kikuchi, Kumamoto | LC573915, LC586108 | - |  |
| 50 | KH12P-1 | + | − | + | 2012 | Haramizu, Kikuyo, Kikuchi, Kumamoto | LC573916, LC586109 | - |  |
| 51 | KH12P-2 | − | − | − | 2012 | Haramizu, Kikuyo, Kikuchi, Kumamoto | - | - |  |
| 52 | YH12P-1 | + | − | + | 2012 | Yatsushiro, Kumamoto | - | - |  |
| 53 | YH12P-2 | − | − | − | 2012 | Yatsushiro, Kumamoto | - | - |  |
| 54 | T2-1 | + | − | − | 2012 | Miyao, Oita, Oita | LC573917, LC586110 | - |  |
| 55 | T2-2 | − | − | + | 2012 | Miyao, Oita, Oita | - | - |  |
| 56 | T4-1 | + | − | − | 2012 | Fukura, Oita, Oita | - | - |  |
| 57 | T4-2 | + | − | + | 2012 | Fukura, Oita, Oita | LC573918, LC586111 | LC573944 |  |
| 58 | T6-1 | − | − | − | 2012 | Kamihetsugi, Oita, Oita | - | - |  |
| 59 | T6-2 | − | − | − | 2012 | Kamihetsugi, Oita, Oita | - | - |  |
| 60 | T8-1 | − | − | − | 2012 | Nozu, Usuki, Oita | - | - |  |
| 61 | T8-2 | − | − | − | 2012 | Nozu, Usuki, Oita | - | - |  |
| 62 | H1-2 | − | − | + | 2012 | Shibayama, Chitose, Bungo-ono, Oita | - | - |  |
| 63 | S1-1 | + | − | + | 2012 | Nagayu, Naoiri, Taketa, Oita | LC573919, LC586112 | - |  |
| 64 | S1-2 | − | − | − | 2012 | Nagayu, Naoiri, Taketa, Oita | - | - |  |
| 65 | MZ1-12-1 | − | − | + | 2012 | Kamino, Takachiho, Nishiusuki, Miyazaki | - | LC573945 |  |
| 66 | MZ1-12-2 | − | − | − | 2012 | Kamino, Takachiho, Nishiusuki, Miyazaki | - | - |  |
| 67 | MZ2-12-1 | − | − | − | 2012 | Hinokage, Nishiusuki, Miyazaki | - | - |  |
| 68 | MZ2-12-2 | + | − | + | 2012 | Hinokage, Nishiusuki, Miyazaki | LC573920, LC586113 | LC573946 |  |
| 69 | MZ3-12-1 | − | − | − | 2012 | Saigo, Misato, Higashiusuki, Miyazaki | - | - |  |
| 70 | MZ3-12-2 | − | − | − | 2012 | Saigo, Misato, Higashiusuki, Miyazaki | - | - |  |
| 71 | MZ4-12-1 | + | − | + | 2012 | Tsunoda, Kitakata, Nobeoka, Miyazaki | LC573921, LC586114 | - |  |
| 72 | MZ4-12-2 | + | − | + | 2012 | Tsunoda, Kitakata, Nobeoka, Miyazaki | LC573906 | LC573947 |  |
| 73 | MZ9-12-1 | − | − | − | 2012 | Yamada, Miyakonojo, Miyazaki | - | - |  |
| 74 | MZ9-12-2 | + | − | + | 2012 | Yamada, Miyakonojo, Miyazaki | - | LC573948 |  |
| 75 | MZ10-12-1 | − | − | + | 2012 | Yamanokuchi, Miyakonojo, Miyazaki | - | LC573949 |  |
| 76 | MZ12-12-1 | + | − | + | 2012 | Mimata, Kitamorokata, Miyazaki | LC573922, LC586115 | LC573950 |  |
| 77 | MZ12-12-2 | + | − | − | 2012 | Mimata, Kitamorokata, Miyazaki | - | - |  |
| 78 | MZ13-12-1 | + | − | + | 2012 | Kitago, Nichinan, Miyazaki | LC573907 | - |  |
| 79 | MZ13-12-2 | + | − | + | 2012 | Kitago, Nichinan, Miyazaki | LC573923, LC586116 | LC573951 |  |
| 80 | MZ15-12-1 | + | − | + | 2012 | Nojiri, Kobayashi, Miyazaki | - | LC573952 |  |
| 81 | MZ15-12-2 | − | − | + | 2012 | Nojiri, Kobayashi, Miyazaki | - | - |  |
| 82 | MZ17-12-1 | + | − | − | 2012 | Saigo, Misato, Higashiusuki, Miyazaki | LC573924, LC586117 | - |  |
| 83 | MZ17-12-2 | + | − | + | 2012 | Saigo, Misato, Higashiusuki, Miyazaki | LC573925, LC586118 | LC573953 |  |
| 84 | MZ19-12-1 | + | − | + | 2012 | Kunitomi, Higashimorokata, Miyazaki | LC573926, LC586119 | LC573954 |  |
| 85 | MZ19-12-2 | + | − | − | 2012 | Kunitomi, Higashimorokata, Miyazaki | LC573927, LC586120 | - |  |
| 86 | KMK12P-1 | − | − | + | 2012 | Ono, Kinpo, Mianamisatsuma, Kagoshima | - | - |  |
| 87 | KMK12P-2 | − | − | + | 2012 | Ono, Kinpo, Mianamisatsuma, Kagoshima | - | - |  |
| 88 | KMK12P-3 | − | − | + | 2012 | Ono, Kinpo, Mianamisatsuma, Kagoshima | - | - |  |
|  | **Strain** | **Viruses** | | | **Year** | **Location, prefecture** | **Accession no.** | |  |
|  |  | **MoV2** | **MoCV1** | **MoPV1** |  |  | **MoV2** | **MoPV1** |  |
| 89 | KMK12P-4 | − | − | + | 2012 | Ono, Kinpo, Mianamisatsuma, Kagoshima | - | - |  |
| 90 | KMK12P-5 | − | − | + | 2012 | Ono, Kinpo, Mianamisatsuma, Kagoshima | - | LC573955 |  |
| 91 | KMK12P-6 | − | − | + | 2012 | Ono, Kinpo, Mianamisatsuma, Kagoshima | - | - |  |
| 92 | KIO12P-1 | + | − | + | 2012 | Okuchi, Isa, Kagoshima | LC573928, LC586121 | LC573956 |  |
| 93 | KIO12P-2 | + | − | + | 2012 | Okuchi, Isa, Kagoshima | LC573929, LC586122 | - |  |
| 94 | KIO12P-3 | + | − | + | 2012 | Okuchi, Isa, Kagoshima | LC573930, LC586123 | LC573957 |  |
| 95 | KIO12P-4 | + | − | + | 2012 | Okuchi, Isa, Kagoshima | LC573931, LC586124 | - |  |
| 96 | KIO12P-5 | + | − | + | 2012 | Okuchi, Isa, Kagoshima | LC573932, LC586125 | LC573958 |  |
| 97 | KIO12P-11 | + | − | + | 2012 | Okuchi, Isa, Kagoshima | LC573933, LC586126 | LC573959 |  |
| 98 | KIO12P-19 | − | − | − | 2012 | Okuchi, Isa, Kagoshima | - | - |  |
| 99 | KIO12P-20 | − | − | − | 2012 | Okuchi, Isa, Kagoshima | - | - |  |
| 100 | KIO12P-21 | − | − | − | 2012 | Okuchi, Isa, Kagoshima | - | - |  |
| **Hokuriku region** | | | | | | | | |  |
| 1 | 01150-01 | − | − | − | unknown | Takada, Agano, Niigata | - | - |  |
| 2 | 01150-02 | − | − | − | unknown | Norikiyo, Shibata, Niigata | - | - |  |
| 3 | 01150-03 | − | − | − | unknown | Obiori, Sanjo, Niigata | - | - |  |
| 4 | 01150-06 | − | − | + | unknown | Iida, Sanjo, Niigata | - | - |  |
| 5 | 01150-12 | − | + | + | unknown | Warimachi-shinden, Kariwa, Kariwa, Niigata | - | - |  |
| 6 | 01150-15 | − | − | + | unknown | Takada, Agano, Niigata | - | - |  |
| 7 | 01150-18 | − | + | − | unknown | Yokaichi, Murakami, Niigata | - | - |  |
| 8 | 01150-19 | − | − | − | unknown | Arasawa, Murakami, Niigata | - | - |  |
| 9 | 01150-20 | − | − | + | unknown | Sambusho, Ojiya, Niigata | - | - |  |
| 10 | 01150-21 | − | + | + | unknown | Shimojima, Uonuma, Niigata | - | - |  |
| 11 | 01150-22 | − | − | − | unknown | Yoshimizu, Uonuma, Niigata | - | - |  |
| 12 | 01150-25 | − | + | − | unknown | Nagamatsu, Minami-uonuma, Niigata | - | - |  |
| 13 | 01150-27 | − | + | + | unknown | Urasa, Minami-uonuma, Niigata | - | - |  |
| 14 | 01150-28 | − | + | + | unknown | Ikazuchi, Minami-uonuma, Niigata | - | - |  |
| 15 | 01150-29 | − | − | + | unknown | Myogasawa, Minami-uonuma, Niigata | - | - |  |
| 16 | 01150-30 | − | − | − | unknown | Amagashima-shinden, Minami-uonuma, Niigata | - | - |  |
| 17 | 01150-31 | − | + | − | unknown | Izumi-shinden, Minami-uonuma, Niigata | - | - |  |
| 18 | 01150-32 | − | − | − | unknown | Shitoka, Minami-uonuma, Niigata | - | - |  |
| 19 | 01150-34 | − | + | + | unknown | Yamaguchi, Minami-uonuma, Niigata | - | - |  |
| 20 | 01150-35 | − | − | − | unknown | Yamaguchi, Minami-uonuma, Niigata | - | - |  |
| 21 | 01150-37 | − | + | + | unknown | Ozuki, Minami-uonuma, Niigata | - | - |  |
| 22 | 01150-38 | − | − | − | unknown | Koguriyama, Minami-uonuma, Niigata | - | - |  |
| 23 | 01150-39 | − | − | + | unknown | Kimizawa, Minami-uonuma, Niigata | - | - |  |
| 24 | 01150-40 | − | − | − | unknown | Date, Tokamachi, Niigata | - | - |  |
| 25 | 01150-41 | − | + | − | unknown | Komichiyama, Tokamachi, Niigata | - | - |  |
| 26 | 01150-42 | − | − | − | unknown | Toriyama, Tokamachi, Niigata | - | - |  |
| 27 | 01150-44 | − | − | − | unknown | Shimofunato, Tsunan, Naka-uonuma, Niigata | - | - |  |
| 28 | 01150-45 | + | − | − | unknown | Shimofunato, Tsunan, Naka-uonuma, Niigata | - | - |  |
| 29 | 01150-46 | − | − | − | unknown | Nakafukami, Tsunan, Naka-uonuma, Niigata | - | - |  |
| 30 | 01150-47 | − | − | − | unknown | Shimofunato, Tsunan, Naka-uonuma, Niigata | - | - |  |
| 31 | 01150-48 | − | − | − | unknown | Sambusho, Ojiya, Niigata | - | - |  |
| 32 | 01150-49 | − | − | − | unknown | Kowada, Ojiya, Niigata | - | - |  |

|  | **Strain** | **Viruses** | | | **Year** | **Location, prefecture** | **Accession no.** | |
| --- | --- | --- | --- | --- | --- | --- | --- | --- |
|  |  | **MoV2** | **MoCV1** | **MoPV1** |  |  | **MoV2** | **MoPV1** |
| 33 | 01150-50 | − | − | + | unknown | Shimojima, Uonuma, Niigata | - | - |
| 34 | 01150-52 | − | + | + | unknown | Oura-Shinden, Uonuma, Niigata | - | - |
| 35 | 01150-53 | − | − | + | unknown | Oura, Uonuma, Niigata | - | - |
| 36 | 01150-55 | − | − | + | unknown | Itagi, Uonuma, Niigata | - | - |
| 37 | 01150-56 | − | − | + | unknown | Nagamatsu, Uonuma, Niigata | - | - |
| 38 | 01150-57 | − | − | + | unknown | Obiroo, Uonuma, Niigata | - | - |
| 39 | 01150-58 | − | − | − | unknown | Obiroo, Uonuma, Niigata | - | - |
| 40 | 01150-59 | − | − | − | unknown | Uonuma, Niigata | - | - |
| 41 | 01150-60 | − | − | + | unknown | Myogasawa, Minami-uonuma, Niigata | - | - |
| 42 | 01150-61 | − | − | − | unknown | Amagashima-shinden, Minami-uonuma, Niigata | - | - |
| 43 | 01150-62 | − | − | − | unknown | Serida, Minami-uonuma, Niigata | - | - |
| 44 | 01150-63 | − | − | − | unknown | Shitoka, Minami-uonuma, Niigata | - | - |
| 45 | 01150-66 | − | − | + | unknown | Ubasawa-Shinden, Minami-uonuma, Niigata | - | - |
| 46 | 01150-68 | − | − | + | unknown | Date, Tokamachi, Niigata | - | - |
| 47 | 01150-70 | + | − | − | unknown | Komichiyama, Tokamachi, Niigata | - | - |
| 48 | 01150-71 | − | − | − | unknown | Maibara, Tsunan, Naka-uonuma, Niigata | - | - |
| 49 | 01150-74 | − | + | + | unknown | Kiyosato, Joetsu, Niigata | - | - |
| 50 | 01150-75 | − | − | + | unknown | Minamigata, Joetsu, Niigata | - | - |
| 51 | 01150-76 | − | − | − | unknown | Yamaya, Kakizaki, Joetsu, Niigata | - | - |
| 52 | 01150-78 | − | − | + | unknown | Shinanozaka, yasuzuka, Joetsu, Niigata | - | - |
| 53 | 01150-79-1 | − | + | + | unknown | Amamizukoshi, Matsunoyama, Tokamachi, Niigata | - | - |
| 54 | 01150-87 | − | − | − | unknown | Ono, Itoigawa, Niigata | - | - |
| 55 | 01150-92 | − | − | + | unknown | Fukuro, Sanjo, Niigata | - | - |
| 56 | 01160-20 | − | − | + | unknown | Kume, Himi, Toyama | - | - |
| 57 | 01160-22 | − | − | + | unknown | Nakano, Imizu, Toyama | - | - |
| 58 | 01160-25 | + | − | + | unknown | Higashifuse, Kurobe, Toyama | - | - |
| 59 | 01160-26 | − | − | + | unknown | Shitadan, Tateyama, Naka-niikawa, Toyama | - | - |
| 60 | 01160-27 | − | − | + | unknown | Takano, Tateyama, Naka-niikawa, Toyama | - | - |
| 61 | 01160-30 | − | + | + | unknown | Shimomura, Fuchu, Toyama, Toyama | - | - |
| 62 | 01160-33 | − | − | + | unknown | Yasuchi, Yatsuo, Toyama, Toyama | - | - |
| 63 | 01160-34 | − | − | + | unknown | Shimbo, Toyama, Toyama | - | - |
| 64 | 01160-37 | − | − | + | unknown | Ofuse, Kurobe, Toyama | - | - |
| 65 | 01170-01-1 | − | − | − | unknown | Nishiyachi, Nakajima, Nanao, Ishikawa | - | - |
| 66 | 01170-02 | − | − | − | unknown | Sugimori, Nanao, Ishikawa | - | - |
| 67 | 01170-05 | − | − | − | unknown | Nishiyachi, Nakajima, Nanao, Ishikawa | - | - |
| 68 | 01170-06 | − | − | + | unknown | Shitsumi, Anamizu, Hosu, Ishikawa | - | - |
| 69 | 01170-07 | − | − | + | unknown | Takigahara, Komatsu, Ishikawa | - | - |
| 70 | 01170-09 | − | − | − | unknown | Nishiyachi, Nakajima, Nanao, Ishikawa | - | - |
| 71 | 01170-10 | − | − | − | unknown | Jike, Hakui, Ishikawa | - | - |
| 72 | 01170-11 | − | − | − | unknown | Takigahara, Komatsu, Ishikawa | - | - |
| 73 | 01170-12 | − | − | + | unknown | Matsunagi, Suzu, Ishikawa | - | - |
| 74 | 01170-13 | − | − | − | unknown | Shitsumi, Anamizu, Hosu, Ishikawa | - | - |
| 75 | 01170-14 | − | − | + | unknown | Shitsumi, Anamizu, Hosu, Ishikawa | - | - |
| 76 | 01180-09 | − | − | + | unknown | Kawai, Fukui, Fukui | - | - |
| 77 | 01180-10 | − | − | + | unknown | Eihei, Yoshida, Fukui | - | - |
| 78 | 01180-17 | − | + | + | unknown | Noune, Maruoka, Sakai, Fukui | - | - |
|  | **Strain** | **Viruses** | | | **Year** | **Location, prefecture** | **Accession no.** | |
|  |  | **MoV2** | **MoCV1** | **MoPV1** |  |  | **MoV2** | **MoPV1** |
| 79 | 01180-18 | − | − | − | unknown | Shinjo, Sakai, Sakai, Fukui | - | - |
| 80 | 01180-19 | − | − | − | unknown | Yubinaka, Arawa, Fukui | - | - |
| 81 | 01180-20 | − | − | + | unknown | Kunugi, Arawa, Fukui | - | - |
| 82 | 01180-23 | − | − | − | unknown | Minami-yokoji, Maruoka, Sakai, Fukui | - | - |
| 83 | 01180-25 | − | − | − | unknown | Kunugi, Arawa, Fukui | - | - |
| 84 | 01180-26 | − | + | − | unknown | Yubinaka, Arawa, Fukui | - | - |
| 85 | 01180-28 | − | − | − | unknown | Higashi-tanaka, Arawa, Fukui | - | - |
| 86 | 01180-29 | − | − | + | unknown | Kawai, Fukui, Fukui | - | - |
| 87 | 01180-30 | − | + | + | unknown | Okabo, Fukui, Fukui | - | - |
| 88 | 01180-32 | − | − | − | unknown | Morida, Fukui, Fukui | - | - |
| **Other region** | | | | | | | | |
| 1 | APU10-199A | + | + | + | 2010 | Akita | - | - |
| 2 | Yamagata_2013 | + | + | + | 2014 | Yamagata | LC573937, LC586127 | LC573960 |
| 3 | IB10 | + | − | + | 2010 | Ibaraki | LC573934 | - |
| 4 | IB11 | + | − | + | 2010 | Ibaraki | LC573935 | - |
| 5 | IB12 | + | − | + | 2010 | Ibaraki | LC573936 | - |
| 6 | Ken 60-19 | + | − | − | 1960 | Aichi | - | - |

^a^The name of host *M. oryzae* strain*.*

^b^Mycovirus positive pattern detected by dsRNA electrophoresis assay indicates “+” and negative indicates “−”

^c^Year when *M. oryzae* strain was collected.

^d^Location where the *M. oryzae* strain was collected in Japan.

^e^Accession number of complete or partial nucleotide sequence determined in this study.

**Supplementary Table S3.** Population genetic parameters for the full length of RdRp and CP sequences of five MoV2 isolates.

| **Virus** | **Gene** | ***n***^a^ | **Net sites** | ***S*** | ***h*** | **π** | **Hd** | ***d*_N_** | ***d*_S_** | ***d*_N_/*d*_S_** |
| --- | --- | --- | --- | --- | --- | --- | --- | --- | --- | --- |
| MoV2 | RdRp | 5 | 2493 | 207 | 5 | 0.04420 | 1.000 | 0.00634 | 0.14010 | 0.04525 |
| MoV2 | CP | 5 | 2366 | 233 | 5 | 0.05135 | 1.000 | 0.00443 | 0.17344 | 0.02554 |

^a^Five isolates of MoV2 (Accession no.) were used: Ken 60-19 (AB300379), APU10-199A (LC432343), NHH12P-1 (LC573905), MZ4-12-2 (LC573906), and MZ13-12-1 (LC573907).

Net sites, length of sequence (excluding sites with gaps and missing data); *S*, number of polymorphic sites; *h*, number of haplotypes; π, nucleotide diversity; Hd, haplotype diversity; *d*_N_, nonsynonymous rate; *d*_S_, synonymous rate.

**Supplementary Table S4.** Property of the determined MoV2 complete genome.

| MoV2 isolate^a^  (Accession no.) | |  | 5'UTR | ORF1 | ORF2 | 3'UTR | whole | overlapped sequence^b^ |
| --- | --- | --- | --- | --- | --- | --- | --- | --- |
| NHH12P-1  (LC573905) | nt  aa | | 275 | 2367  788 | 2493  830 | 63 | 5194 | AUGA |
| MZ4-12-2  (LC573906) | nt  aa | | 275 | 2367  788 | 2493  830 | 63 | 5194 | AUGA |
| MZ13-12-1  (LC573907) | nt  aa | | 275 | 2367  788 | 2496  831 | 63 | 5197 | AUGA |

^a^Indicates host *M. oryzae* strain names.

^b^Overlapped nucleotide sequence between start codon of ORF2 and terminal codon of ORF1.
